# New glacier evidence for ice-free summits during the life of the Tyrolean Iceman

**DOI:** 10.1038/s41598-020-77518-9

**Published:** 2020-12-17

**Authors:** Pascal Bohleber, Margit Schwikowski, Martin Stocker-Waldhuber, Ling Fang, Andrea Fischer

**Affiliations:** 1 Institute for Interdisciplinary Mountain Research, Austrian Academy of Sciences, Innsbruck, Austria; 2 Department of Environmental Sciences, Informatics and Statistics, Ca’Foscari University of Venice, Venice, Italy; 3 Paul Scherrer Institute, Villigen, Switzerland; 4 Department for Chemistry and Biochemistry, University of Bern, Bern, Switzerland; 5 Oeschger Center for Climate Change Research, University of Bern, Bern, Switzerland

**Keywords:** Cryospheric science, Palaeoclimate

## Abstract

Detailed knowledge of Holocene climate and glaciers dynamics is essential for sustainable development in warming mountain regions. Yet information about Holocene glacier coverage in the Alps before the Little Ice Age stems mostly from studying advances of glacier tongues at lower elevations. Here we present a new approach to reconstructing past glacier low stands and ice-free conditions by assessing and dating the oldest ice preserved at high elevations. A previously unexplored ice dome at Weißseespitze summit (3500 m), near where the “Tyrolean Iceman” was found, offers almost ideal conditions for preserving the original ice formed at the site. The glaciological settings and state-of-the-art micro-radiocarbon age constraints indicate that the summit has been glaciated for about 5900 years. In combination with known maximum ages of other high Alpine glaciers, we present evidence for an elevation gradient of neoglaciation onset. It reveals that in the Alps only the highest elevation sites remained ice-covered throughout the Holocene. Just before the life of the Iceman, high Alpine summits were emerging from nearly ice-free conditions, during the start of a Mid-Holocene neoglaciation. We demonstrate that, under specific circumstances, the old ice at the base of high Alpine glaciers is a sensitive archive of glacier change. However, under current melt rates the archive at Weißseespitze and at similar locations will be lost within the next two decades.

## Introduction

The majority of the glacier ice volume in the Alps may disappear within the next two centuries^1^. A major scientific question is whether this process of deglaciation is unprecedented within the Holocene. If we want to assess the pace of future deglaciation in the Alps, it is essential to understand past glacier dynamics and their relation to changes in climate^2^. We already have comprehensive information about the maximum extent of Alpine glaciers from the investigation of moraine positions and ages^3,4^. Yet comparatively little is known so far about the times of minimum ice cover or ice-free conditions at high-elevation. Indeed, only the dating of wood fossil emerged from ice melt has made it possible to constrain glacier retreat, but at lower elevation^5,6^. Complementary information to findings from glacier tongues can come from glaciers at high elevations^7^, where the ice is frozen to bedrock. Despite the strong retreat since the Little Ice Age (LIA), access to the oldest ice near bedrock is still complicated for high-elevation areas and usually requires drilling glacier ice cores.

Most previous ice core research in the Alps was aimed at continuous stratigraphic climate records. This limited it to glaciers where there is usually no melting on the surface throughout the year. Few suitable drilling sites exist as they are mostly confined to above 4000 m altitude, e.g. locations in the Western Alps^8^. In the Eastern Alps and at elevations below 4000 m, such records are sparse, with only one ice core record obtained from an already partially temperate site at Ortler^9^ and at the temperate Silvretta glacier^10^. Still, it generally holds that if basal ice temperatures are below the melting point, the so-called “cold ice” remains frozen to bedrock. If it moves at all, it does so by slow internal deformation, in contrast to temperate ice (i.e. ice at around 0 °C), which is able to slide along the bedrock^11^. Even though its remaining thickness is only around 10 m, millennial-old stagnant cold ice has recently been found at the base of an Alpine summit glacier^12^. Constraining the maximum age of the stagnant ice near bedrock can indicate past ice-free periods, followed by neoglaciation^13^. Hence this age information alone contains important information on paleoclimatic conditions.

Until recently, only human artifact findings were discussed in connection with low stands of high-elevation glaciers in the Alps; at Schnidejoch pass (2750 m, Bernese Alps^7^) and the “Tyrolean Iceman” in the Eastern Alps^14^. Schnidejoch pass is easily blocked by glacier advances, making the artifact findings a sensitive indicator of past glacier minima. Radiocarbon dating of artifacts indicates three phases of minimal ice extent, suitable for crossing the pass. The earliest phase was around 6.8–6.3 ka cal, a second phase from 5.7 to 4.9 ka cal, and an adjacent third period from 4.9 to 4.2 ka cal^15^. The Tyrolean Iceman mummy emerged from a small ice field, vanished since, at Tisenjoch, a saddle located at 3210 m. Radiocarbon dating of the mummy indicated that the Iceman lived roughly 5.1–5.3 ka cal^14,16^. The well-preserved state of the corpse and of artifacts suggests that they had been conserved in frozen conditions. The ice field at Tisenjoch must therefore have been present during several known periods of glacier retreat, such as the Roman and Medieval warm phases^17^. Unfortunately, after the discovery of the Iceman, pollen analyses were only conducted on the surrounding ice^18^. With the arrival of modern radiometric ice-dating techniques based on ^14^C-dating of the water-insoluble organic carbon fraction of carbonaceous aerosols embedded in the ice matrix, it is now possible to constrain the age of the glacier ice itself^19,20^. Dating the ice with today’s radiometric techniques could have told us if the Iceman had in fact died in a mostly ice-free environment, or if he fell into a crevasse on a glacier-covered Tisenjoch.

## Results

### The unique site for old ice preservation at Weißseespitze

**Figure 1:**
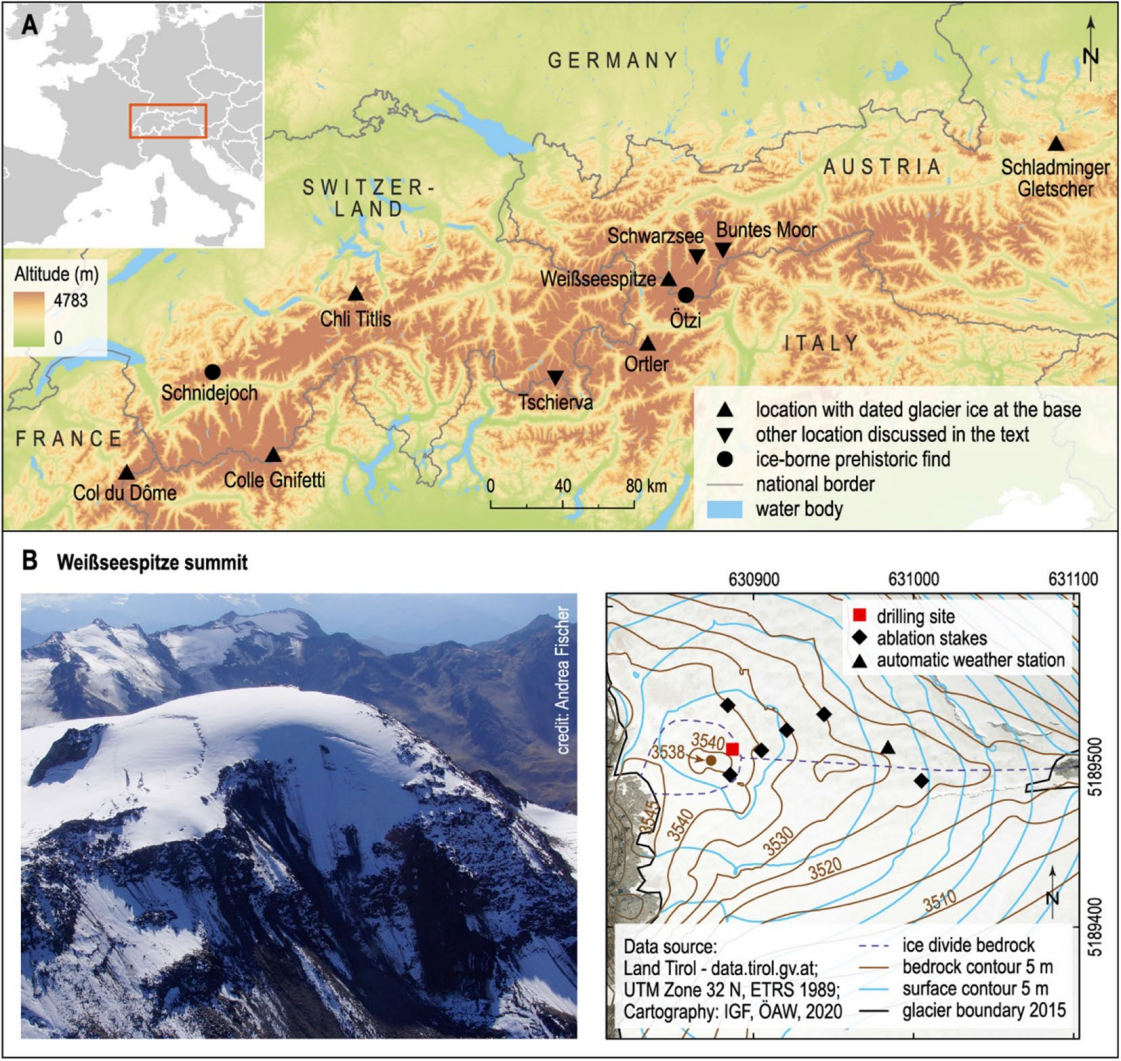
The location of the Weißseespitze summit glacier, the discovery site of the Tyrolean Iceman (“Ötzi”) and other sites discussed in the text (**A**). Shown in the bottom row is a close up of the glacier topography at Weißseespitze summit and the precise drilling location (**B**). Note the unique dome shape of the summit glacier. Data sources part A: EuroBoundaryMap, copyright EuroGeographics; CCM River and Catchment Database copyright European Commission—JRC, 2007; CIAT-CSI SRTM (http://srtm.csi.cgiar.org); Cartography: IGF, ÖAW, 2020; under a CC-BY-NC-SA 4.0 International License (https://creativecommons.org/licenses/by-nc-sa/4.0/). Maps made with licensed software Arc Map 10.6.1 (https://desktop.arcgis.com/en/arcmap/), figure made with licensed software Adobe Illustrator CS6 (https://www.adobe.com).

The Weißseespitze summit glacier (WSS, 3500 m) marks the highest point of Gepatschferner in the Austrian Alps. It is a unique site: (1) located only 12 km from the Iceman location, it offers a potential surrogate to investigate the local glacier conditions during the lifetime of the Iceman; (2) it has a dome-shaped glacier geometry, which is an extremely rare feature in the Alps (Fig. 1). Historical photographs dating back to about 1888, maps and digital elevation models, as well as direct measurements at the summit ice dome reveal continuous volume loss since the LIA maximum (Table 1) around 1855 for Gepatschferner^21^. The limited ice thickness and dome geometry mean minimal to no ice flow, confirmed by differential GPS measurements at stakes in 2018 and 2019. In these circumstances, the influence of ice dynamics at the ice divide is negligible for ice age interpretation. Despite the ablation measured at stakes in 2018 and 2019, englacial borehole temperatures remained permanently sub-zero at 1 m below surface, with − 3 °C at 9 m depth, implying that the ice is frozen to bedrock (“Methods” section).

**Table 1:** Elevation change as ice loss indicator at Weißseespitze summit, given in metres per year and derived from digital elevation models (DEM, previous studies) and direct measurements by ablation stakes (this work).

Period	Elevation change at drilling site (DoD [m])				References
	ΔH/year		DEM	Vert. accuracy (m)	
2018–1969	− 0.29		1969	± 1.9	^29^
2018–1997	− 0.20		1997	± 0.7	^29^

	Ablation at stake(m)				
	ΔH/year				
2018	− 0.88	± 0.02			This work
2019	− 0.63	± 0.02			This work

*DoD* difference of DEMs.

### Ice core analysis and glacier age constraints

**Figure 2:**
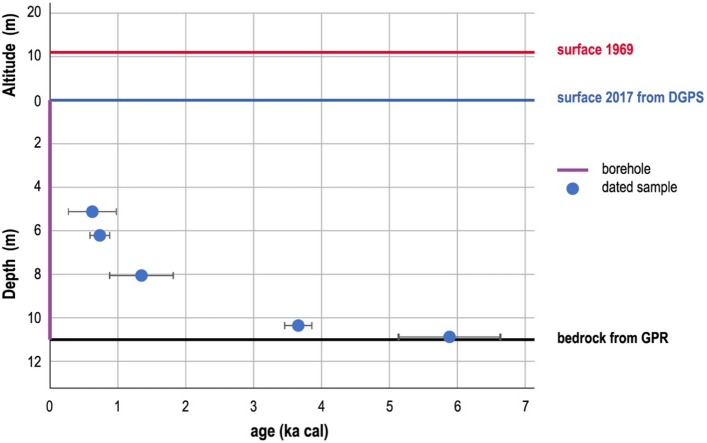
Depth-age relation of the Weißseespitze ice core, obtained by aerosol-based micro-radiocarbon dating of the ice. Also shown is the reconstructed surface elevation loss between 1969 and 2017. Figure made with licensed software Adobe Illustrator CS6 (https://www.adobe.com).

In March 2019, two parallel cores were drilled 11 m to bedrock at the ice divide with nearly flat bed conditions. Visible layers of refrozen meltwater indicate that there was only limited occasional melt at this site when this ice was formed. The main part of the ice core includes bubble-rich glacier ice, the likely result of dry metamorphosis of snow. This view is supported by stable oxygen (δ^18^O) and hydrogen (δD) ratios of a range typical for the seasonal variation in snow at this altitude, with no deviation from the meteoric water line (“Methods” section). Within the upper 4 m of ice, a tritium content in the order of 100 mBq/kg suggests that the ice was formed before widespread tritium release from nuclear weapons tests in the early 1960s (maximum concentrations in 1963 are expected to exceed 10 Bq/kg) and that the layers from that time had already been removed by melting. The present surface at WSS is thus older than 1963, with the remaining ice the remnant of an originally thicker ice cap. The aerosol-based micro-^14^C dating of five samples from the ice core returned ages continuously increasing with depth, from (0.62 ± 0.35) ka cal (i.e. calibrated years in ka before 1950, 1 sigma range) at about 4.5 m depth to (5.884 ± 0.739) ka cal just above the bed (Fig. 2 and “Methods” section). All ages are reported in the text on this timescale in ka. The original dates are found in the given references.

## Discussion

We evaluated the new evidence from Weißseespitze, first, in view of existing information regarding the regional climate history, followed by the pan-Alpine context of ice-dated glacier minima. For the latter, we paid special attention to co-evaluating maximum age and elevation of all sites.

Despite the drastic mass loss on the surface in today’s conditions, the basal ice was found to remain frozen to the bed. Our dating of the ice just above bedrock indicates that the ice body at WSS formed earlier than (5.9 ± 0.7) ka cal and has been glaciated continuously ever since. This implies that even the WSS summit location at 3500 m altitude was ice-free during an interval prior to (5.9 ± 0.7) ka. In the context of other regional climate evidence, this finding is consistent with the glacier length reconstructions of Gepatschferner, which document a distinct minimal extent starting around 5.9 ka^21^. Likewise, at around 5.3–5.1 ka cal, no ice existed at nearby Tisenjoch, at 3210 m^22^. The fact that the lifetime of the Iceman falls within the maximum age range determined for the WSS summit glacier suggests that a rather rapid neoglaciation ended the formerly near ice-free conditions at the summits in this region.

The end of the so-called “Holocene Climatic Optimum”^14,23^ is also observed in Austrian stalagmite records, indicating the onset of a cooling period around 5.9 ka^24^. In the Eastern Alps, tree-ring-dated subfossil wood remains indicate that several advances occurred between 5.9 and 5.5 ka at three different glaciers in the Alps: Unteraar, Pasterze, Tschierva^25^. The former two have much longer response times than Tschierva, and generally fluctuations of glacier tongues deviate from mass balances at summit glaciers because the terminus position also depends on ice dynamics and because wind erosion affects net accumulation at summits. Nonetheless, the general picture agrees well. Around 5.9 ka, there was a limited advance at Tschierva glacier, reaching the 2000 ice extent and ending the preceding warm period^25^. During those warm periods, the tree line was up to 165 m above the 1980 tree line in Kaunertal, in ultimate vicinity to WSS^26^. A chironomid record obtained at Schwarzsee in nearby Oetztal suggests that roughly between 5.2 and 4.5 ka cal a climate transition occurred, with a distinct cooling trend in summer temperatures, which prevailed until the end of the LIA^27^.

**Figure 3:**
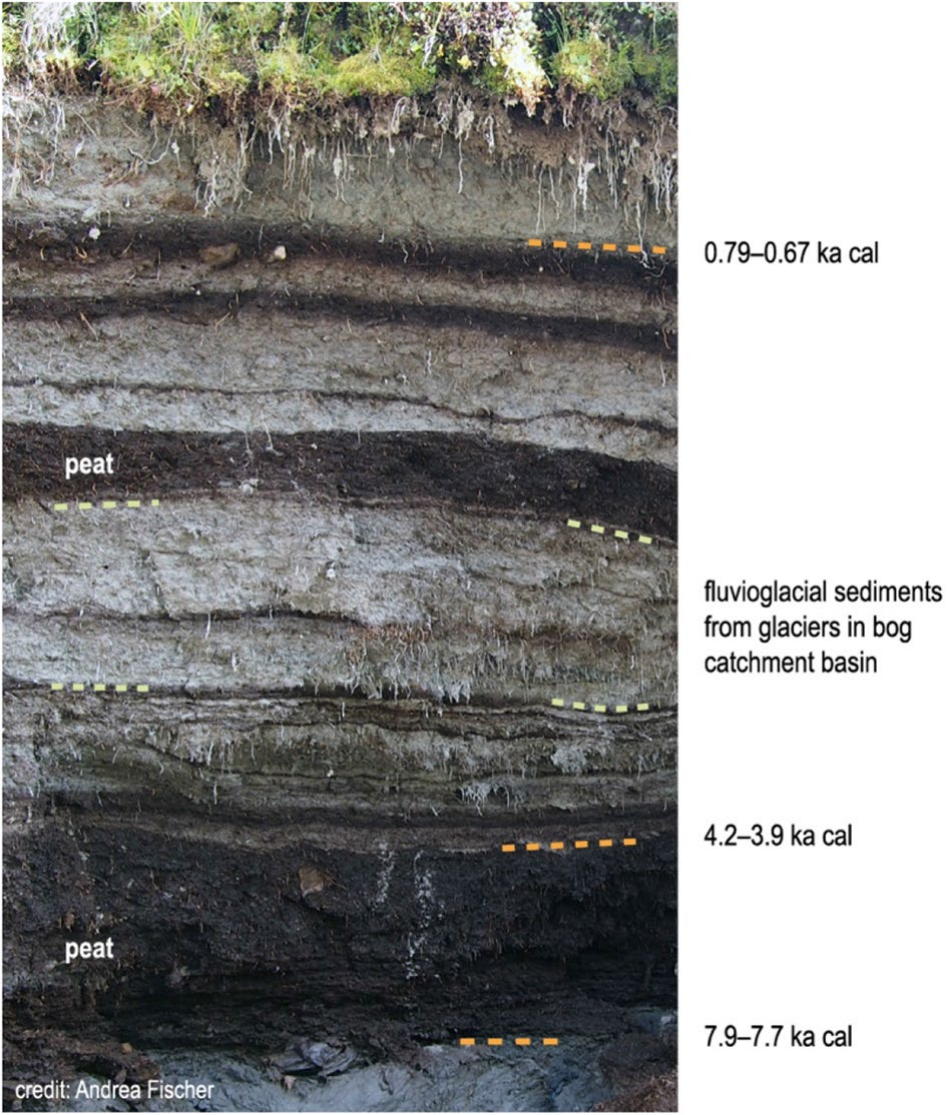
Photograph of the Buntes Moor bog profile with indicated radiocarbon ages^28^ as orange dashed lines. Buntes Moor is located contiguous to the LIA terminus of Fernauferner glacier. Grey layers are fluvioglacial sediments from the basin (e.g. yellow dashed lines). Dark layers are peat formed during warm periods. Figure made with licensed software Adobe Illustrator CS6 (https://www.adobe.com).

This is consistent with other regional evidence provided by the Oberfernau bog sediments (Buntes Moor): a radiocarbon-dated layer marks the end of peat growth during a warm phase (Fig. 3). From about 4.2–3.9 ka cal onwards, the fluvioglacial sediments indicate the presence of glaciers in the catchment area, i.e. a cooler period^28^. Although the effective altitude of the glaciers leading to the fluvioglacial deposits is not closely defined, it must have been significantly lower than the WSS summit. In this regional context, the findings from WSS fit remarkably well into a general picture of regional warm conditions ending around 5.9 ka at high altitude. This was followed by a period of glacier advance that started around the lifetime of the Iceman, with a delayed onset of glaciation on lower elevation summits.

**Figure 4:**
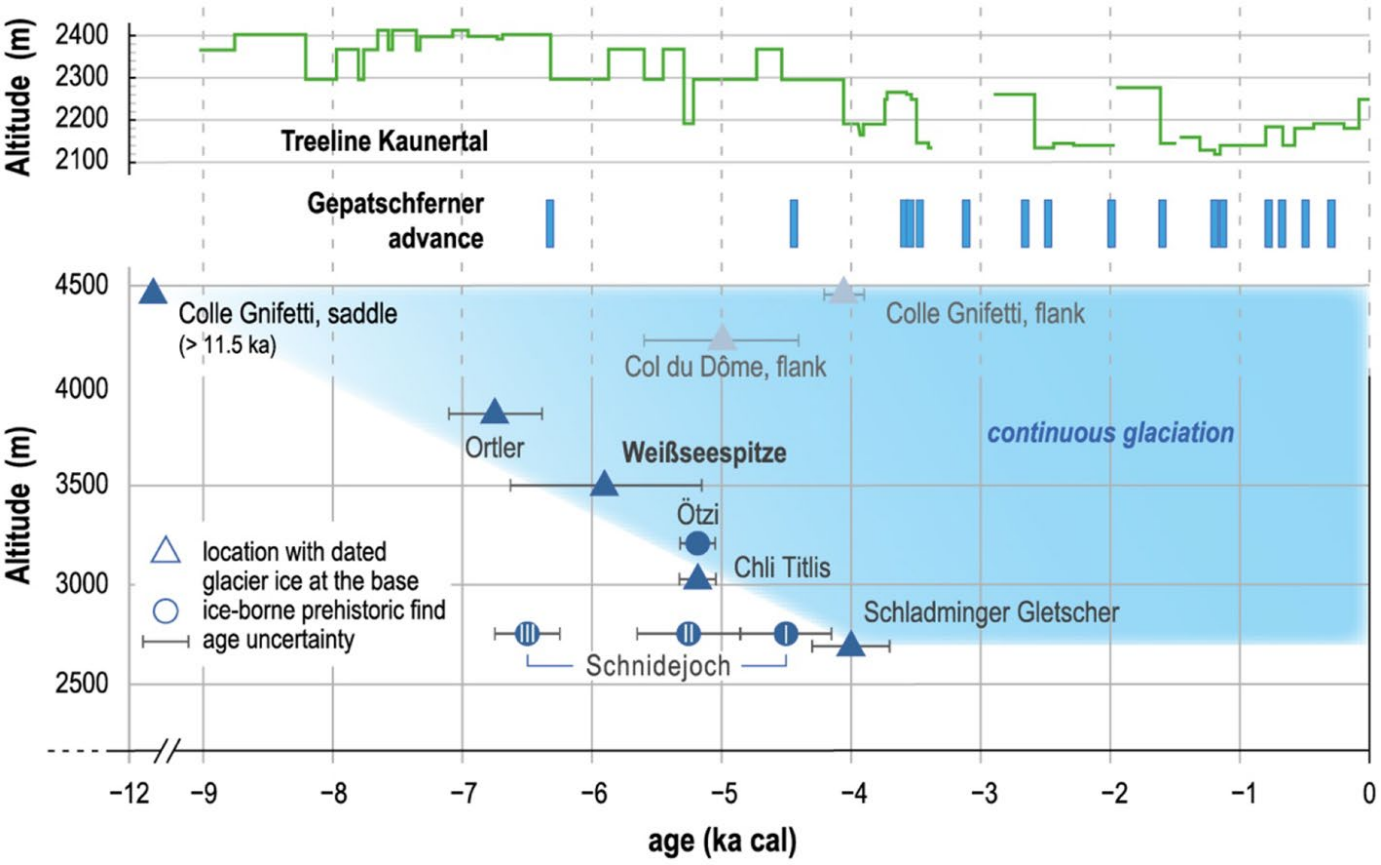
Compilation of dated past neoglaciation events at high-elevation locations in the Alps. Note the general correspondence between glacier maximum age and its altitude. The blue shaded area indicates the period of continuous ice cover. Also shown are the tree line reconstruction from Kaunertal^26^ and the reconstructed advances of Gepatschferner greater than its 1960 extent^21^. References: Colle Gnifetti saddle^19,31^, flank^20^; Col du Dome^32^; Ortler^9^; Iceman “Ötzi”^14,16^; Chli Titlis and Schladminger Glacier^12^; Schnidejoch^15^. Figure made with licensed software Adobe Illustrator CS6 (https://www.adobe.com).

The findings from WSS are integrated in an overview of already existing maximum age constraints (Table 2), in order to further investigate the neoglaciation history of high-elevation glaciers throughout the Alps, and the role of the altitude of the site. As shown in Fig. 4, this compilation reveals a remarkable gradient in the onset of neoglaciation in relation to the altitude of the site. Although the altitude of glacier tongues depends primarily on precipitation, as suggested by today’s glacier distribution in Austria^29^, this may not be true of high Alpine glaciers. At high-elevation and summit locations, glaciers are typically very limited in size and heavily exposed to wind erosion as a main constraint on net accumulation there^8^. Instead, the dependence on altitude found here suggests a primary connection to mean annual temperature, and its lapse rate, for neoglaciation at these sites. In a simplistic view, a cooling trend would lead to snow accumulation, prolonged positive mass balance and subsequent ice formation on highest elevation sites earlier than on lower elevation, as the ablation rate is smaller at the former. Vice versa, warm periods might lead to increased ablation and prolonged negative mass balance on lower sites, leaving higher glaciers unchanged in geometry, as is the case today for the highest summits of the Western Alps^30^.

**Table 2:** Compilation of glacier maximum age constraints.

Site name	Elevation (m)	Maximum age (ka cal)	Uncertainty (ka)	References
Schladminger Gletscher	2700	4.0	0.3	^12^
Schnidejoch I	2756	4.9–4.2	n.a	^15^
Schnidejoch II	2756	5.7–4.9	n.a	^15^
Schnidejoch III	2756	6.8–6.3	n.a	^15^
Chli Titlis	3030	5.2	0.1	^12^
Iceman Oetzi	3210	5.3–5.1	n.a	^14,16^
Weißseespitze	3500	5.9	0.7	This work
Ortler	3859	6.7	0.4	^9^
Col du Dome—flank	4200	5.0	0.6	^32^
Colle Gnifetti—flank	4450	4.1	0.2	^20^
Colle Gnifetti—saddle	4450	> 11.5	n.a	^19,31^

Within the region of WSS, maximum glacier age constraints were obtained from a deep ice core drilled at Ortler summit glacier: aerosol-based micro-^14^C dating indicates a glacier formation starting earlier at the higher altitude of around 3860 m, namely around (6.7 ± 0.4) ka cal^9^. For glaciers above 4000 m, maximum age constraints were obtained from ice cores drilled at Colle Gnifetti (4450 m, Monte Rosa massif) and Col du Dôme (4300 m, Mont Blanc massif). Both glaciers are in the highest region of the glacier’s accumulation zone and feature a saddle-shaped geometry. As opposed to the ice dome at WSS, the saddle geometry affects the interpretation of the ice core dating in relation to glacier maximum age constraints. At Colle Gnifetti, for two ice cores drilled close to the saddle point, radiocarbon dates from the deepest layers point to ice older than 11.5 ka cal^19,31^. In contrast, in an ice core drilled at a flank position, a basal age of only (4.1 ± 0.2) ka cal was obtained^20^. Likewise, for an ice core drilled at a flank position at Col du Dôme, the basal age was constrained recently to about (5.0  ± 0.6) ka cal^32^.

Two non-exclusive interpretations may explain the age difference between saddle and flank locations. Due to ice flow at flank positions, the deepest ice layer originating from the neoglaciation may have been severely thinned, making it difficult to adequately resolve its age in a bulk sample. Moreover, the original neoglaciation may have started as a small ice patch around the saddle and not reached the flanks until a further build-up of the glacier was completed. Notably, if taken at face value as the maximum glacier age, the radiocarbon dates from the saddle positions at Colle Gnifetti are a good fit with the elevation gradient of neoglaciation revealed at sites below 4000 m (Fig. 4).

The elevation-age gradient implies that in the Alps only the highest elevation sites, such as Colle Gnifetti, remained ice-covered throughout the Holocene. This view is corroborated by the fact that the summits above 4000 m show only minor volume changes even under current climate conditions^30^. Regarding the presence of glaciers below 4000 m, Holocene climate variability has sufficed to induce their de- and subsequent neoglaciation. Considering the current ice loss at WSS and elsewhere, a systematic shift towards a warmer future climate will eradicate these ice archives permanently.

Although the spatial density of dated ice archives is particularly high in the case of the Alps, our approach can, in principle, be transferred to other mountain ranges to study their Holocene neoglaciation history. This appears especially promising where age constraints on the deepest layers of high-elevation glaciers already exist^33^. However, the discussion of the maximum age constraints at Colle Gnifetti illustrates the importance of topographic effects for interpreting the fluctuation and presence of glaciers as indicators of climate^34^ and climate change^29^. In this context, it is worth noting that point mass balance data have been shown to reflect changes in climate better than total mass balance or terminus fluctuation^35^. Cold high-elevation glaciers with low ice dynamics, as almost ideal–typical at WSS, thus present a more direct link to past climate change than terminus fluctuations. In addition, the altitude of the site affects the sensitivity of any glaciers to changes in climate. Although the altitudinal target range may be different for every mountain range, we have demonstrated here for the Alps how certain high-elevation glaciers provide valuable information of Holocene glaciation. This provides important background knowledge for planning sustainable development in mountain regions as they approach nearly ice-free conditions in a warmer future climate.

## Conclusion

In a compilation with existing glacier age constraints, the unique ice dome at Weißseespitze has closed a regional and altitudinal gap to reveal the first direct evidence for an elevation gradient of Holocene neoglaciation in the Alps. While only the highest elevation sites remained ice-covered throughout the Holocene, summits around 3000–4000 m were likely ice-free during the Mid-Holocene or covered by glaciers distinctly smaller than today. Around the lifetime of the Tyrolean Iceman and slightly earlier, rapid ice formation started and some of this ice cover exists to this day. Impressive current melt rates threaten the extinction of these ice archives: Weißseespitze glacier, which has accumulated over nearly 6000 years, may disappear within just two decades. However, it may not be the deglaciation of the summits during the Holocene that is unprecedented, but its pace, on which we urgently need extensive empirical information. Our findings demonstrate that the cold ice at certain glaciers, even below 4000 m, is indeed a sensitive archive that delivers a baseline of at least 5 millennia of Alpine glacier change, which allows us not only to tackle past climate variability, but also to find evidence for the effects of climate change mitigation over future decades.

## Methods

### Site characteristics

**Figure 5:**
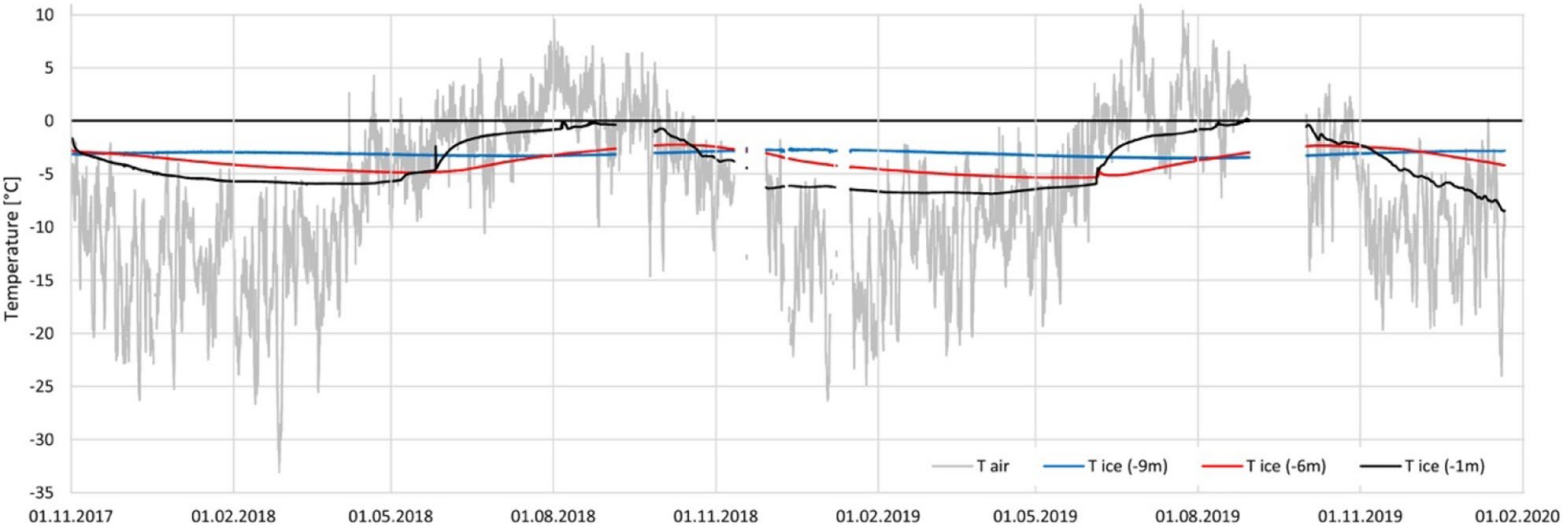
Results from englacial temperatures from the automatic weather station at the WSS summit (grey: air temperature, blue: ice temperature at 9 m depth, red: ice temperature at 6 m depth, black: ice temperature at 1 m depth). Sub-zero temperatures at 9 m depth with a mean temperature of − 3.1 °C for the whole period indicate that the basal ice below is frozen to bedrock, despite air temperatures of up to 11.1 °C inducing strong surface melt. Figure made with licensed software Microsoft Excel 2016 (https://www.microsoft.com/).

The summit of Weißseespitze features a unique ice dome geometry, with its main ice divide running roughly from an ice-free section in the east towards the cliff on its western side. The ice thickness around the ice divide ranges from 6 to 12 m measured with a GSSI SIR 4000 GPR (500 MHz). The total surface elevation change since 1969 exceeds 10 m, based on digital elevation models (Table 1). No presence of a firn layer was found in snow pits apart from seasonal snow coverage. Englacial temperature measurements verify cold-ice conditions and indicate ice frozen to bedrock (Fig. 5). The mean air temperature is − 6.9 °C for the 2-year period 1 November 2017–31 October 2019 (T max: + 11.1 °C, T min: − 33.1 °C). Ice flow velocities at the ice divide were measured at stakes with differential GPS. Within the measurement accuracy of ± 10 cm, no motion was detected between July 2017 and October 2019.

### Ice core drilling and core handling

Two cores were drilled at the same location on WSS down to bed using a 2″ electro-mechanical drill^36^. Cores were packed on site into insulating boxes and transported via cold-storage transport to the cold-room facilities (− 20 °C) of the Institute of Environmental Physics, Heidelberg University. The visual stratigraphy of the ice cores showed white, bubble-rich ice layers, occasionally interrupted by cm-thick clear layers of refrozen meltwater. The lowermost 30 cm of the cores showed small rocks incorporated into the ice, a clear sign of bedrock proximity. This is consistent with scratch marks on the cutters of the core barrel after the last drilling run to bedrock. No further ice could be recovered, providing clear evidence that bedrock had been reached.

### Tritium and stable water isotope analysis

**Figure 6:**
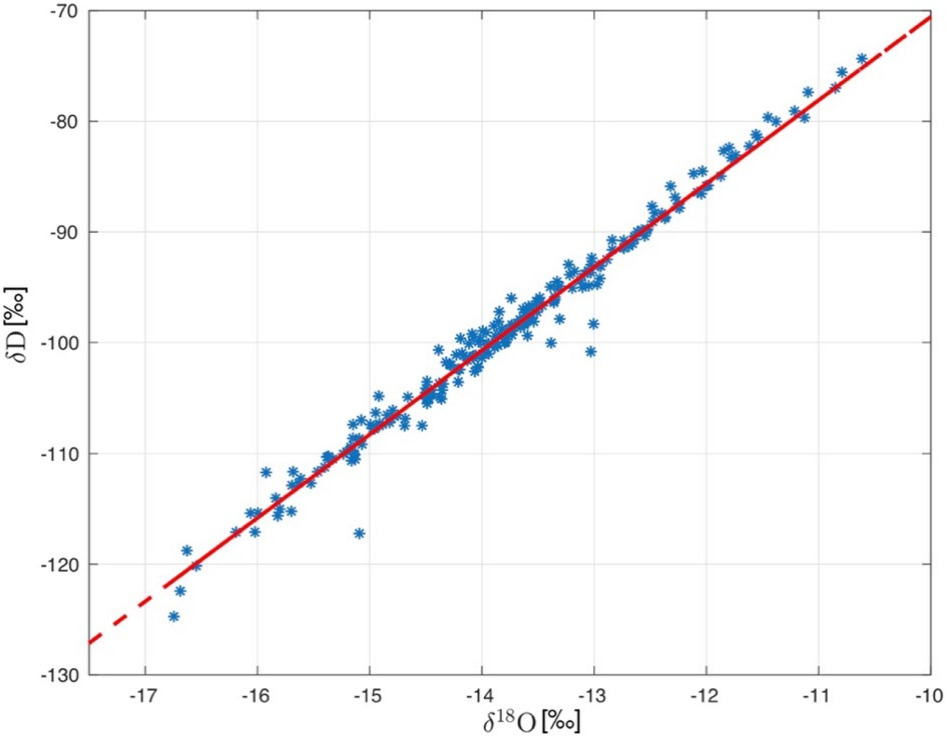
Results from co-isotopic analysis of the WSS ice core. δD and δ^18^O are closely correlated (r = 0.99). Linear regression results in a slope of 7.5, close to the global meteoric water line. Figure made with licensed software MATLAB R2019 (https://www.mathworks.com).

In addition to the deep cores drilled to bedrock, three shallow cores were drilled to 3–4 m depth, at positions around the location of the deep ice core drilling. From the shallow cores, three samples each of 300 ml (i.e. 34 cm length) were prepared from the top, bottom and an intermediate section. The samples were shipped with cold-storage transport to Seibersdorf Laboratories, Seibersdorf, Austria, where tritium was measured after electrolytic enrichment, which was necessary because of the low concentrations (Table 3). From the main ice core drilled to bedrock, subsamples for stable water isotope analysis were cut manually at 5 cm intervals to 8.3 m depth and at 2.5 cm between 8.3 m and the end of the core (11.13 m). Measurements were performed at the Institute of Environmental Physics, Heidelberg University, using a cavity ring-down spectrometer. Measurement uncertainties range within ± 0.1 and ± 0.4‰ for δ^18^O and δD, respectively^12^. Calculating ordinary linear regression of δD against δ^18^O yields a slope of 7.5 at a correlation coefficient of r = 0.99 (Fig. 6). The slope lies close to the global meteoric water line (slope 8), whereas melting and refreezing would lead to reduced values of the slope^37^. Hence, the co-isotopic analysis presents no clear signs of isotopic change after ice formation for the bulk part of the stratigraphy.

**Table 3:** Tritium concentrations of three shallow cores drilled at the WSS summit in 2018.

Sample	Depth range (cm)	Measurement (mBq/kg)	Uncertainty (mBq/kg)	Detection limit (mBq/kg)
Shallow core 1 top	0–34	838	35	47
Shallow core 1 middle	116–147	484	35	47
Shallow core 1 bottom	214–247	< 47	–	47
Shallow core 2 top	0–34	1345	35	59
Shallow core 2 middle	213.5–247.5	602	35	59
Shallow core 2 bottom	454.5–488.5	118	35	59
Shallow core 4 top	0–28	1015	35	47
Shallow core 4 middle	82–114	486	35	47
Shallow core 4 bottom	118–217.5	57	47	47

### Aerosol-based micro-radiocarbon ice dating

A total of six samples selected for micro-radiocarbon dating (55–70 cm in length) was shipped via cold-storage transport to the Paul Scherrer Institute (PSI). There, samples were rinsed with ultrapure water to remove the potentially contaminated outer layer. Around 20–30% of the ice mass was lost in this step. Samples were melted at room temperature in 1L containers (Semadeni, PETG) and filtered with prebaked (5 h at 800 °C) quartz-fiber filters. The Water Insoluble Organic Carbon (WIOC) and Elemental Carbon (EC) fractions were separated using a commercial combustion system (Sunset Laboratory Inc., USA) with a recently developed thermal-optical method (Swiss_4S). The resulting CO_2_ was quantified with a non-dispersive infrared (NDIR) detector at the Laboratory for the Analysis of Radiocarbon with AMS (LARA Laboratory) of the University of Bern. The Sunset instrument is coupled to a gas introduction interface system (GIS), which in turn is coupled to a MIni radioCArbon DAting System (MICADAS; developed at ETH Zurich). The system allows online ^14^C measurements of the carbonaceous fractions separated by the Sunset. ^14^C ages were calibrated using OxCal software with the Northern Hemisphere (IntCal13, no significant changes are observed when using IntCal20) calibration curve^38,39^. The micro-radiocarbon dating results are summarized in the Table 4.

**Table 4:** Micro-radiocarbon dating results.

Core section	Depth (m)	Ice mass (g)	WIOC (µg)	Be no	F^14^C	^14^C age (Cal-BP)
WS7	5.46	397.7	170.4	12,317.1.1	0.930 ± 0.046	623 ± 350
WS9	6.47	251.9	19.8	12,741.1.1	0.910 ± 0.019	735 ± 145
WS12	8.33	434.5	87.8	12,318.1.1	0.847 ± 0.046	1347 ± 456
WS16	10.57	296.1	68.9	12,742.1.1	0.657 ± 0.013	3661 ± 200
WS17-1	11.13	174.9	7.2	11,922.1.1	0.529 ± 0.041	5884 ± 739
WS17-2^a^	Basal ice	151.6	104.9	11,921.1.1	0.223 ± 0.006	14,020 ± 332

^a^Due to visible silt material and high organic carbon content, the nominal age of the lowermost basal sample is biased by reservoir effects. It is reported for completeness but was not considered further for interpretation.
